# Emissivity of Building Materials for Infrared Measurements

**DOI:** 10.3390/s21061961

**Published:** 2021-03-11

**Authors:** Eva Barreira, Ricardo M. S. F. Almeida, Maria L. Simões

**Affiliations:** 1 CONSTRUCT-LFC, Department of Civil Engineering, Faculty of Engineering (FEUP), University of Porto, Rua Dr. Roberto Frias s/n, 4200-465 Porto, Portugal; ralmeida@estv.ipv.pt (R.M.S.F.A.); lurdes.simoes@fe.up.pt (M.L.S.); 2 Polytechnic Institute of Viseu, Campus Politécnico de Repeses, 3504-510 Viseu, Portugal

**Keywords:** emissivity, infrared thermography, emissometer, test procedures, black tape method, moisture content

## Abstract

Infrared thermography (IRT) is a technique increasingly used in building inspection. If in many applications it is sufficient to analyze the thermal patterns, others exist in which the exact determination of the surface temperature is a fundamental aspect. In these circumstances, the emissivity of the surfaces assumes special relevance, being probably the most important property in the definition of the boundary conditions. However, information on the uncertainty involved in its measurement, as well as the conditions that influence it, is scarce. This article presents an innovative contribution both to the characterization of the emissivity of various construction materials, and to the discussion of emissivity measurement procedures and the attendant uncertainty. In this sense, three experimental campaigns were carried out: T.I, preliminary tests to assess the initial conditions required for an accurate IRT measurement of the emissivity (reference tape and position of the camera); T.II, assessment of the emissivity of nine different building materials, in dry conditions, using the emissometer and the IRT and black tape methods; and T.III, assessment of the emissivity of three materials during the drying process. The results confirmed that emissivity is a crucial parameter for the accurate measurement of surface temperature. Emissivity measurements carried out with IRT (black tape method) and with the emissometer returned meaningful differences when compared with the values available in the literature. This disagreement led to surface temperature differences of up to 7 °C (emissometer versus reference values). This research also highlighted that the moisture content of the materials influences the emissivity values, with fluctuations that can be greater than 10%, and that the effect of moisture is visible even for low values of moisture content.

## 1. Introduction

Infrared thermography (IRT) is a technique increasingly used in building inspection. If in many applications it is sufficient to analyze the thermal patterns, others exist in which the exact determination of the surface temperature is a fundamental aspect. Traditional applications of IRT include the detection of insulation heterogeneities [1], air infiltration [2,3], anomalies related to moisture [4,5,6,7,8,9] and thermal bridges [10], the assessment of HVAC systems [11] and construction details [12,13], and the identification of defects in façades [12,13,14]. On the other hand, due to the major concerns about energy consumption, IRT is increasingly being used to assess the energy efficiency of buildings [15,16,17,18,19,20]. 

Emissivity (*ε*) defines the ability of a material to emit energy, and is strongly correlated with its surface characteristics. Emissivity values can vary between 0 (perfect reflector/mirror) and 1 (perfect emitter/blackbody) [21]. This can be defined as the ratio of the radiation emitted by the surface (*R_t_*) to the radiation emitted by a blackbody (*R_t_(blackbody)*) at the same temperature (Equation (1)). The Stefan–Boltzmann law (Equation (2)) calculates the total amount of radiation emitted by the blackbody at a certain temperature *T*, in every direction and over all wavelengths [22].

(1) $$\varepsilon =\frac{R_{t}}{R_{t}\left(blackbody\right)}$$

(2) $$R_{t}=\sigma .T^{4}$$

where *R_t_* is the total blackbody spectral radiance (W/m^2^), *σ* is the Stefan–Boltzmann constant ($\sigma =5.67\times 10^{-8}{\;W/m}^{2}K^{4}$) and *T* is the temperature (K).

The non-blackbody emitters for which the emissivity is constant regardless of the wavelength are called grey bodies. The total amount of radiation emitted by the grey body can be calculated using the Stefan–Boltzmann law and considering the emissivity of the surface, *ε*, as described by Equation (3) [21]. Most materials used in buildings exhibit behavior that is similar to the grey body. For that reason, for these materials, emissivity can be used as a constant for a certain temperature [23].

(3) $$R_{t}=\varepsilon .\sigma .T^{4}$$

A range of emissivity values can be found in the literature, depending on the surface characteristics, temperature and wavelength of the measurement [22,23,24]. An important pioneering piece of research was carried out by Wan et al. [25], in which spectral radiance measurements were made in the laboratory and in the field for deriving the spectral emissivity values of some land cover samples, using a spectroradiometer and an auxiliary radiation source in the wavelength range 2.5–14.5 μm. Many methods have been used over the past few decades to determine emissivity. According to Albatini [26,27], a direct measurement of the radiance reflected by the material can be used to evaluate emissivity. Smetana and Reicher [28] suggested an indirect measurement method that enabled the determination of the normal spectral emissivity of various materials at a specific wavelength. Ianiro and Cardone [29] estimated emissivity through the multi-wavelength pyrometry principle applied to IRT, using two IR cameras in a stereo arrangement with detectors working in different wavelength bands. Gallet et al. [30] measured emissivity by comparing the surface temperature of the sample under study with the equivalent temperature of the blackbody, which was simulated by one system formed from the same sample and a reflective hemisphere that was targeted through an opening. In addition, Mathew et al. [31] used a similar method to assess the emissivity of geological samples. Marinetti and Cesaratto [32] proposed a transient method for measuring emissivity without the use of reference materials, and Ciocia and Marinetti [33] determined the emissivity values of reference labels (black paper, white paper and aluminium) in the laboratory by a dynamic test, and applied them to characterize different building materials in situ.

Avdelidis and Moropoulou [20] and Asdrubali et al. [8] assessed the emissivity values of different materials using the ASTM E 1933—99a standard that provided procedures for measuring the surface temperature of a specimen with contact or noncontact thermometers. In both cases, the devices were calibrated, and the specimen was at a temperature at least 10 °C warmer or cooler than the ambient temperature. The surface emissivity was adjusted by equating the temperature measured with the IR camera to the one measured by the thermometer.

If a portion of the surface under study can be painted with a black paint of known emissivity (about 1.0), the emissivity of the surface can be assessed by adjusting it until the surface temperatures measured on the coated surface and on the surface without coating are the same [34]. A similar procedure was adopted by several authors, although instead of black paint, black electrical tape was used [12,20,35,36,37,38,39]. This method is based on the determination of the emissivity of materials by comparing their optic characteristics with the ones of a nearby blackbody at the same temperature, simulated by the black paint or tape. In fact, combining Equations (1)–(3), it is possible to determine the emissivity of the surface when the blackbody’s (black paint or tape) temperature and emissivity are known.

Nowadays, two widespread standards are used to assess the emissivity of a material: (i) the ASTM standard E1933—14 [40], which provides guidelines to measure the emissivity of materials using infrared imaging systems; and (ii) the standard ASTM C1371—04a 41, which defines the procedures for the determination of the emittance of materials near room temperature using a portable emissometer.

The literature review shows that there is no consensus on the procedure for determining the emissivity of construction materials. Therefore, evaluating the advantages and limitations of practical procedures to determine the surface’s emissivity that can be applicable in situ is very important, as no information on their accuracy is available in the literature. When one intends to assess the emissivity of materials in situ, in addition to the obvious limitations associated with the method itself (conditioned access to the site, positioning of the equipment, etc.), the boundary conditions associated with the properties of the material can also play an important role. In this context, the moisture content of the materials is a property that should be considered, especially since it is a value that can vary significantly over the material’s lifecycle, thus introducing additional uncertainty into the measurements.

This article presents an innovative contribution both to the characterization of the emissivity of various construction materials and to the discussion of the emissivity measurement procedures. In this sense, the results of several experimental campaigns with specific objectives are presented, namely:

- Assess the initial conditions required for an accurate IRT measurement of the emissivity;
- Evaluate the reliability of the black tape procedure by comparing its results with the ones given by measurements made with an emissometer and with reference values available in the literature;
- Analyze and discuss the effect of the material’s moisture content on the emissivity.

## 2. Methodology

### 2.1. Test Procedures 

#### 2.1.1. General Considerations

This experimental campaign was divided into three main tasks:

- T.I: Preliminary tests to assess the initial conditions required for an accurate IRT measurement of the emissivity, namely:
  ○ T.Ia: The effect of the reference tape in the measurements when applying the black tape procedure. The emissivity of nine commercial tapes, with different colors and brightnesses, was measured using the emissometer in accordance with the ASTM C1371—04a standard [41]. Although commonly only black tapes are used, it was decided to extend the measurements to other colors to verify if they could also be considered acceptable as a reference tape. The importance of the tape’s brightness was also tested;
  ○ T.Ib: The effect of the position of the camera (angle of the view 45°, 90° and 135°) on the measurement of the emissivity. To that end, ten specimens of ceramic brick (in dry conditions) were used to compare the emissivity measured by means of the emissometer [41] and by the IRT and black tape method [40].
- T.II: Assessment of the emissivity of nine different building materials, in dry conditions, using the emissometer [41] and the IRT and the black tape method [40]. The obtained results were also compared with the reference values available in the literature [42]. The main objective of these tests was to evaluate the accuracy of the black tape method to determine the emissivity of materials by comparison with the results given by the emissometer;
- T.III: Assessment of the emissivity of three materials during the drying process. The specimens were immersed in water until a constant mass was reached, and afterward the emissometer was used to measure the emissivity [41] during the drying process. The main objective of these tests was to evaluate the effect of moisture content on the emissivity of the materials.

In these three tasks, all the measurements were repeated three times, and no relevant variations in air temperature, relative humidity and velocity occurred in the laboratory while they were carried out.

#### 2.1.2. Measurements Using the Emissometer

**Figure 1:**
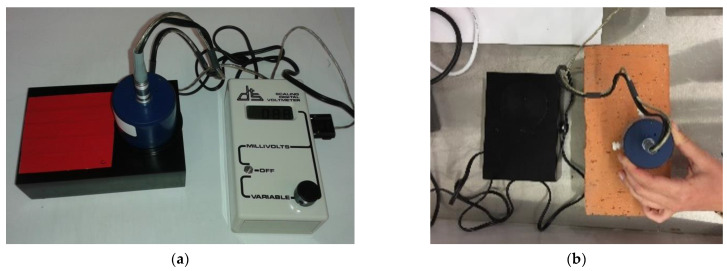
Test set up to assess the emissivity using the emissometer: (**a**) task T.Ia; (**b**) tasks T.II and T.III.

To assess the emissivity of the tapes (T.Ia), the methodological approach of the ASTM C1371—04a standard [41] was followed (Figure 1a). To assess the emissivity of the building materials (T.II and T.III), the procedure described in the standard was adapted according to the operation manual of the equipment [43]. In these tests, each specimen was placed on a flat surface near the heat sink, and thermal equilibrium was achieved before the measurement was carried out, using a cooling fan. The emissivity was measured using the slide method that consists of sliding the detector through the surface of the material in predefined time-steps until the detector reading no longer increases upon changing locations. The specimens were large enough to move the detector to an unheated area as many times as necessary (Figure 1b).

#### 2.1.3. Measurements Using IRT and the Black Tape Method

**Figure 2:**
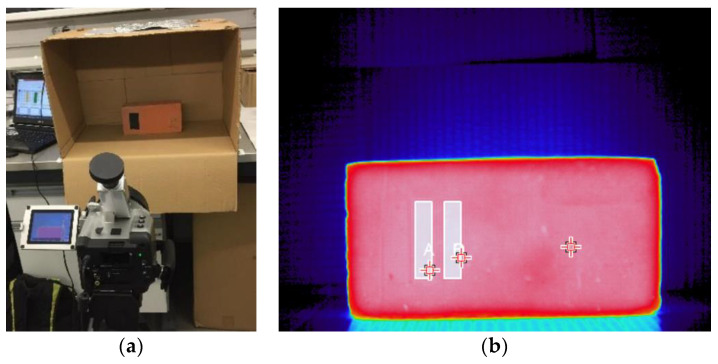
Infrared thermography (IRT). Test set up to assess the emissivity using IRT during tasks T.Ib and T.II: (**a**) measurement; (**b**) processing the measurement results.

To assess the materials’ emissivity in tasks T.Ib and T.II, the specimens were heated over 24 h. Afterwards, each specimen was placed inside a cardboard box to minimize the influence of reflections (Figure 2a). A black matte non-cloth tape (tape A from Table 1), with known emissivity, was used as the reference. Using the camera software, the tape temperature was measured considering its emissivity. The temperature value was the average of the tape temperatures inside a predefined area. The surface emissivity was then adjusted by equating the average temperature of the tape and the average temperature of the surface near it (Figure 2b), considering a similar area [8,20,34,40]. 

### 2.2. Materials

The development of each task in this investigation required the definition of a different set of materials. To assess the emissivity of the tapes (T.Ia), nine different commercial products, with different colors and brightnesses, were selected (Table 1). For task T.Ib, which sought to evaluate the effect of the position of the camera (angle of view) on the emissivity value, a specimen of solid brick was used. To assess the accuracy of the black tape method in determining the emissivity in situ (T.II), nine different materials commonly applied in buildings were selected, and the effect of moisture in the emissivity value (T.III) was evaluated using three different materials. Table 2 described the properties of the materials used in tasks T.Ib, T.II and T.III. These values were obtained from the technical specifications of the materials. 

### 2.3. Equipment

**Figure 3:**
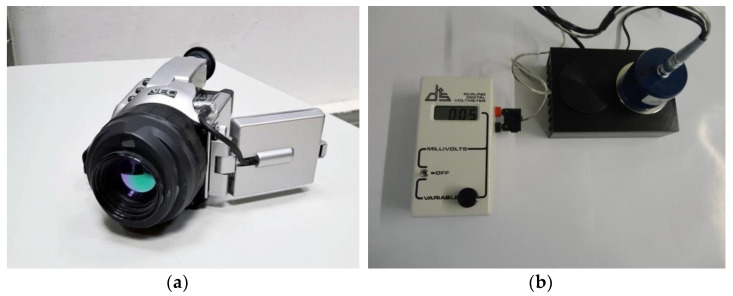
Equipment used in the experimental campaign: (**a**) IR camera; (**b**) emissometer.

In the experimental campaigns, an IR camera (Figure 3a) and an emissometer (Figure 3b) were used. Regarding the IR camera, it was adjusted to compensate the effects of reflection, distance and atmospheric conditions. The parameters introduced for the adjustments were emissivity, ambient temperature, relative humidity, apparent reflected temperature, and distance to target. The apparent reflected temperature was always previously determined using the procedure defined in the standard ASTM E1862—97 (2010) [45]. All thermal images were assessed using the software provided by the camera manufacturer. The basic properties of the IR camera are shown in Table 3.

The emissometer uses a differential thermopile, which comprises one thermopile that is covered with a black coating and one that is covered with a reflective coating, for total hemispherical emissivity measurements. The detector thermopiles are heated in order to provide the necessary temperature difference between the detector and the surface. The equipment is calibrated using two standard samples, one with a high emissivity and the other with a low emissivity, which are placed on the heat sink. The accuracy of the measured values is ±0.02. The emissometer output is 2.4 millivolts nominal, with sample emittance of 0.90 and sample temperature of 25 °C. The detector output is linear with emittance to within ±0.01 units, and the time constant is 10 s, nominal (time to reach 63% of final value) [43].

## 3. Results

### 3.1. Task T.I—Preliminary Tests to Assess the Initial Conditions Required for an Accurate IRT Measurement of the Emissivity

#### 3.1.1. Task T.Ia—Emissivity of the Tapes

Table 4 displays the average (Ave) value of the emissivity of nine (A to I) commercial tapes (three measurements carried out for each tape), which are quite similar as their standard deviations (SD) are small. Regarding the average values of emissivity, they are also very similar, varying between 0.86 (tape G) and 0.89 (tapes F, H and I). The results also show that the color of the tapes does not considerably affect the emissivity value (tapes B, C, D and E, all bright vinyl electrical tapes). In addition, the brightness of the tapes has little impact on the results, although matte tapes present the highest emissivity values (tapes A, F, H and I).

A preliminary statistical analysis was performed using the SPSS software, resulting in the values shown in Table 5. In order to compare the emissivity values obtained for the nine tapes, the Kruskal–Wallis test was applied with a significance level of 5%. The Kruskal–Wallis test compares several populations, in which each one has independent random samples. This test performs a one-way analysis of variance setting. The null hypothesis is that the distribution of the samples is the same among all populations (they have the same average), and the alternative hypothesis is that responses are larger in some populations (at least one has a different average) [46,47,48].

The results (*p*-values are presented in Table 5) show that the emissivity of the tapes cannot be considered statistically identical (*p*-values < 5%) if all the tapes are included. However, if only the matte tapes or only the bright ones (regardless of the color) are considered in the analysis, then the results show that each set is statistically identical for a 5% significance level. This homogeneity of the sample is more evident in matte tapes, as the *p*-value is higher.

Generally, the results allow us to conclude that, when all tapes are assessed, at least one of them has a different emissivity value. On the other hand, if the tape characteristics are identical (matte or bright), the emissivity values are similar. Although the obtained results exhibit little variability, they point to lower values than those usually found in the literature (0.90 to 0.95). These differences point to the importance of an initial assessment of the emissivity of the tape to be used in the measurement if accurate and reliable quantitative IRT is intended. 

#### 3.1.2. Task T.Ib—The Effect of the Angle of View in the Measurement of the Emissivity Using IRT

The emissivity of the solid brick measured with the emissometer and IRT with different angles is shown in Table 6 (average of three measurements for each specimen and method). Similar results were obtained by the two methods, 0.88 for the emissometer and 0.87 for IRT, which are in accordance with the ones found in the literature (Table 2). However, a greater variability occurred when IRT was used, as the coefficients of variation (CV) for all positions of the IR camera were greater than 1.5%, compared to the 0.36% obtained when the emissometer was used.

The small effect of the camera position was already expected, as previous studies showed that angles below 60° do not influence the measurement in non-metallic materials [23]. In this sense, when the test conditions imply angles greater than 60°, an additional preliminary assessment must be carried out.

### 3.2. Task T.II—Emissivity of Different Materials Using the Emissometer and IRT

Table 7 displays the values obtained with the emissometer and the IR camera (three measurements for each material and method). The values obtained with the two methods are quite similar. The only exception is the stainless steel. As already expected, the low emissivity and high reflectivity of this material affects the IRT measurements. Comparing the measured values (from the emissometer and IRT) with the reference ones [42], there are some discrepancies. 

Table 8 shows the relative differences between the values measured with the emissometer and IRT, which average 9.8% if the stainless steel is considered. However, if that material is excluded, the average decreases to 3.2%. If the measured values are compared with the reference ones [42], the average of the differences, without the effect of the stainless steel, increases to 6.4% for the emissometer and to 7.6% for IRT. These differences highlight the importance of measuring emissivity, since the material characteristics and the measurement conditions and procedures may influence the results. Similar conclusions have already been reported by other authors [22].

To highlight the importance of an accurate emissivity measurement, Table 9 shows the surface temperatures of the specimens for different values of emissivity, namely, that obtained with the emissometer, that measured with IRT, and the reference value. The absolute differences between each temperature, and the reference one obtained considering the emissivity measured with the emissometer, are also included. The temperature values differ on average 4.3 °C (ε_IRT_ vs. ε_emissometer_), with a maximum of 29.1 °C for stainless steel and a minimum of 0.4 °C for cork, mortar and untreated wood. The stainless steel is clearly an outlier. If it is not taken into consideration, the average of the differences decreases to 1.3 °C. The differences between temperatures obtained with **ε_IRT_** and **ε_Ref_** are on average 2.1 °C, with a maximum of 7.0 °C for cork and a minimum of 0.0 °C for stainless steel and solid brick.

The importance of these differences in the measurement depends on the aim of the thermographic study. If the measurement of absolute surface temperature is the aim and a quantitative IRT analysis is intended, these differences are very relevant and can bias the conclusions. In building inspection, several examples can be found wherein anomalies can be detected by small temperature differences, sometimes bellow 1 °C [49].

### 3.3. Task T.III—The Effect of Moisture on the Emissivity of Materials

**Figure 4:**
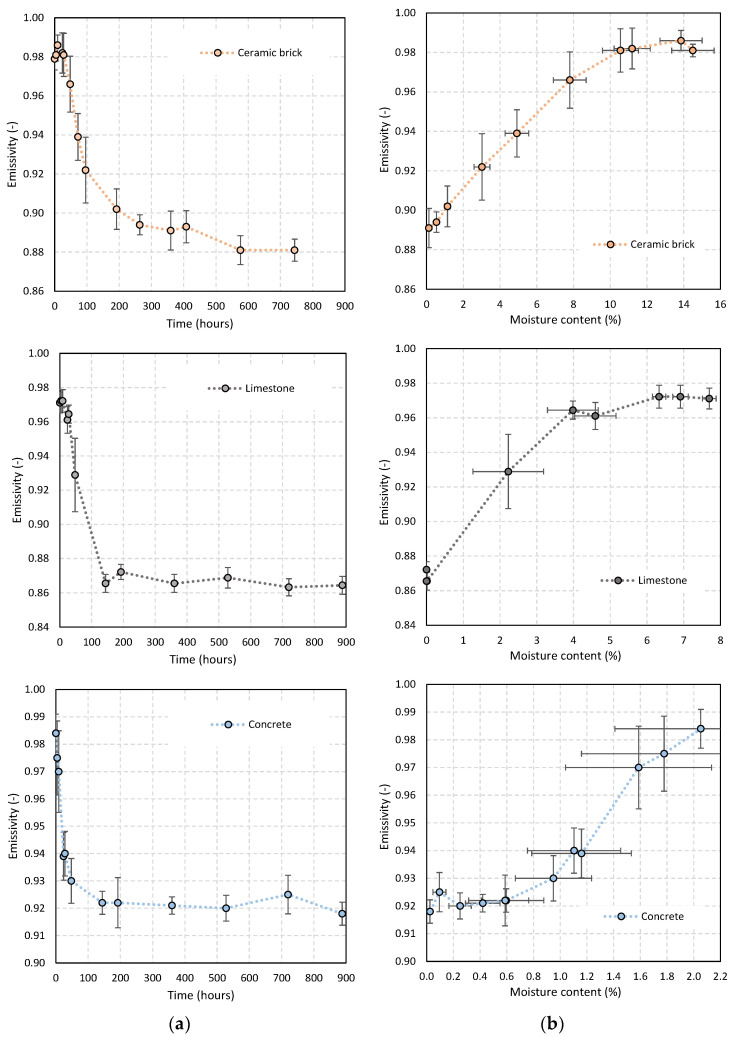
(**a**) Emissivity versus time; (**b**) emissivity versus moisture content.

Construction materials undergo important moisture content variations throughout their lifecycle, which may influence the emissivity of the material. To assess that effect, emissivity was measured during the drying process of ten specimens of three different materials: ceramic brick, limestone and concrete. Figure 4a shows the emissivity variation throughout the drying process, and the relationship between emissivity and moisture content (ratio between the amount of water and the dry weight) is exposed in Figure 4b. To increase the robustness of the analysis, besides the average value, the standard deviation is also included in the graphs.

The effect of humidity on the emissivity measurement is evident from all the graphs, with fluctuations that can be greater than 10%. The fluctuation trend is identical in the three materials, with emissivity values close to 1.0 at the beginning of the drying process (maximum moisture content). With analyzing the drying process, it appears that in the case of ceramic brick and limestone, there is a short initial period (less than 24 h) in which this value is still stable (phase I). After this period, the emissivity begins to decrease. In the case of concrete, the initial stability period is not noticeable, and a decrease in emissivity is immediately observed. The period in which the emissivity reduction is observed accompanies the drying of the material (phase II), after which the emissivity stabilizes and remains approximately constant (phase III). The results for the limestone are the best example from which to clearly observe these three phases during drying, and their relationship with the moisture content of the material.

The transition zone (phase II) clearly portrays a period of greater instability, which is also evident in the greater dispersion of the measurements, in contrast to what happens after the completion of drying, in which the variability of emissivity is considerably reduced. Although observable in all materials, once again, the results for limestone are a good example of this phenomenon.

The results confirm that emissivity is affected by moisture, as it rapidly increases with moisture content, and the effect is visible even for low values of moisture content (higher than 2% in the case of ceramic brick and limestone, and 1.5% in the case of concrete).

## 4. Conclusions

The emissivity of the surface under study is one of the key parameters in the measurements using IRT. It is known that the use of an inaccurate value may influence the results and bias the conclusions, especially when a quantitative analysis is required. However, there are still some uncertainties related to the emissivity measurement procedures and the characterization of the emissivity of construction materials. The literature review revealed that the information on the uncertainty involved in emissivity measurement, as well as the conditions that influence it, is still scarce.

The results of the first experimental work show that the emissivity of the commercial tapes that were assessed cannot be considered statistically identical. However, if the tapes’ brightness values are similar (matte or bright), the emissivity values are similar. The results also show that the values obtained are lower than the ones usually found in the literature, which may justify a preliminary evaluation of the emissivity of the tape to be used in the measurement when quantitative IRT is intended.

The second experimental campaign compared the emissivity measured by the emissometer and IRT with the camera in different positions (45°, 90° and 135°). The average results were similar: 0.88 with the emissometer and 0.87 with IRT. Although some variability occurred in the measurements with IRT, the results confirmed that the assessed angles, below 60°, do not influence the results. However, if the test conditions imply greater angles, an additional preliminary evaluation must be considered.

The third experimental campaign focused on the accuracy of the black tape method in determining emissivity using IRT, by comparing it with measurements carried out with an emissometer. The tests were performed on several materials currently used in buildings, and the results point to the feasibility of the method, as the obtained values are quite similar to the ones given by the emissometer. The only exception was found when assessing the emissivity of metallic materials with high reflection. However, when the measured values are compared with the ones found in the literature, greater differences can be found, which was already expected, as the material characteristics and the measurement conditions and procedures were certainly different. These differences highlight the importance of measuring the emissivity of the surface under analysis before the thermographic assessment begins, although the aim of the study may decrease or increase the importance of deriving an accurate value of the emissivity. Indeed, if a quantitative IRT analysis is intended, the differences found in this work between the measured and the literature values are very relevant and can bias the conclusions. In fact, differences in the measurement of the surface temperature up to 7 °C were found (emissometer versus reference values).

This research also highlighted that the moisture content of the materials influences the emissivity values, with fluctuations that can be greater than 10%. Three phases can clearly be identified during the drying process for the three materials under study. During phase I, corresponding to higher moisture content, the emissivity remains stable and close to 1.0. In phase II, the reduction in the emissivity is consistent with the drying of the material, and a greater variability of the results can be found. Finally, emissivity stabilizes and remains approximately constant (phase III). The effect of moisture is visible even for low values of moisture content (higher than 2% in the case of ceramic brick and limestone, and 1.5% in the case of concrete).

Future works include performing additional laboratory tests using temperatures typically found inside buildings, ranging from 18 to 25 °C, and validation tests carried out in situ.

## Figures and Tables

**Table 1:** Characteristics of the commercial tapes used in task T.Ia.

Ref.	Description
A	Matte black vinyl electrical tape (Scotch^®^ 3M + 33 Super)
B	Bright black vinyl electrical tape (TemflexTM 1300)
C	Bright red vinyl electrical tape (TemflexTM 1300)
D	Bright white vinyl electrical tape (TemflexTM 1300)
E	Bright blue vinyl electrical tape (TemflexTM 1300)
F	Matte black vinyl repairing tape (tesa^®^ Multi Tape)
G	Bright black duct tape (tesa^®^ extra Power Universal)
H	Matte black cloth tape (tesa^®^ extra Power Perfect)
I	Matte black vinyl electrical tape (tesa^®^ iso tape)

**Table 2:** Characteristics of the materials used in tasks T.Ib, T.II and T.III [42,44].

Material	T.Ib	T.II	T.III	Density kg/m^3^	Conductivity W/(m·K)	Specific Heat Capacity J/(kg·K)	Emissivity -
ceramic tile		•		2300	1.3	700	0.92
concrete			•	2200	2.00	880	0.93
cork		•		400	0.07	1700	0.70
granite		•		2600	2.80	750	0.85
gypsum		•		850	0.40	840	0.90
limestone		•	•	2000	1.70	900	0.94
mortar		•		1900	1.30	930	0.90
solid brick	•	•	•	1800	0.45	920	0.88
stainless steel		•		7900	17.0	480	0.16
untreated wood		•		610	0.18	2000	0.85

**Table 3:** Main specifications of the IR camera 42.

Measuring range	–20 °C to 100 °C
Resolution	0.06 °C at 30 °C, 60 Hz
Accuracy	±2 °C or ±2%
Detector	FPA (microbolometer)
Spectral range	8 and 14.0 μm
I.F.O.V	1.2 mrad
Thermal resolution	320 × 240 pixels
Field of view	22° × 16°

**Table 4:** Emissivity values of the nine tapes under study.

Tape	A	B	C	D	E	F	G	H	I
Ave	0.88	0.87	0.87	0.87	0.88	0.89	0.86	0.89	0.89
SD	0.010	0.000	0.010	0.006	0.006	0.010	0.006	0.006	0.010

**Table 5:** *p*-Values of Kruskal–Wallis test to compare the independent samples.

All Tapes	Matte Tapes	Bright Tapes
0.016	0.305	0.187

**Table 6:** Emissivity values obtained with the emissometer and IRT (three different angles).

Method	Emissometer	IRT 45°	IRT 90°	IRT 135°
Ave	0.88	0.87	0.87	0.87
SD	0.003	0.014	0.013	0.017
CV (%)	0.4	1.6	1.5	2.0

**Table 7:** Emissivity measured with the emissometer and IRT, and reference values.

Material	Emissometer	IRT	Reference Values
ceramic tile	0.87	0.82	0.92
cork	0.84	0.85	0.70
granite	0.87	0.81	0.85
gypsum	0.79	0.82	0.90
limestone	0.87	0.83	0.94
mortar	0.93	0.92	0.90
solid brick	0.88	0.87	0.88
stainless steel	0.16	0.06	0.16
untreated wood	0.84	0.85	0.85

**Table 8:** Relative differences between emissivity values obtained with different methods (emissometer, IRT and manual mode of the IR camera).

Material	Emis. vs. IRT	Emis. vs. Ref	IRT vs. Ref
ceramic tile	5.7%	5.7%	12.2%
cork	1.2%	16.7%	17.6%
granite	6.9%	2.3%	4.9%
gypsum	3.8%	13.9%	9.8%
limestone	4.6%	8.0%	13.3%
mortar	1.1%	3.2%	2.2%
solid brick	1.1%	0.0%	1.1%
stainless steel	62.5%	0.0%	166.7%
untreated wood	1.2%	1.2%	0.0%

**Table 9:** Surface temperature measured with IRT (emissivity with the emissometer, IRT and the camera in manual mode) and absolute differences between the temperatures.

Material	Surface Temperature (Absolute Differences Regarding the Temperature Obtained with ε_Emissometer_) (°C)		
	ε_Emissometer_	ε_IRT_	ε_Ref_
ceramic tile	65.9	68.1 (2.2)	63.9 (2.0)
cork	63.8	63.4 (0.4)	70.8 (7.0)
granite	67.5	70.3 (2.8)	68.4 (0.9)
gypsum	68.0	66.6 (1.4)	63.2 (4.8)
limestone	68.1	70.0 (1.9)	65.2 (2.9)
mortar	66.3	66.7 (0.4)	67.6 (1.3)
solid brick	71.6	72.1 (0.5)	71.6 (0.0)
stainless steel	43.9	73.0 (29.1)	43.9 (0.0)
untreated wood	61.0	60.6 (0.4)	60.6 (0.4)
